# Fully Integrated, Stretchable, Wireless Skin‐Conformal Bioelectronics for Continuous Stress Monitoring in Daily Life

**DOI:** 10.1002/advs.202000810

**Published:** 2020-06-15

**Authors:** Hojoong Kim, Yun‐Soung Kim, Musa Mahmood, Shinjae Kwon, Nathan Zavanelli, Hee Seok Kim, You Seung Rim, Fayron Epps, Woon‐Hong Yeo

**Affiliations:** ^1^ George W. Woodruff School of Mechanical Engineering Institute for Electronics and Nanotechnology Georgia Institute of Technology Atlanta GA 30332 USA; ^2^ School of Intelligent Mechatronics Engineering Sejong University Seoul 05006 Republic of Korea; ^3^ Department of Mechanical Engineering University of South Alabama Mobile AL 36608 USA; ^4^ Nell Hodgson Woodruff School of Nursing Emory University Atlanta GA 30322 USA; ^5^ Wallace H. Coulter Department of Biomedical Engineering Parker H. Petit Institute for Bioengineering and Biosciences Institute for Materials Neural Engineering Center Institute for Robotics and Intelligent Machines Georgia Institute of Technology and Emory University Atlanta GA 30332 USA

**Keywords:** continuous stress monitors, galvanic skin response (GSR), stretchable bioelectronics, temperature monitoring, wireless soft electronics

## Abstract

Stress is one of the main causes that increase the risk of serious health problems. Recent wearable devices have been used to monitor stress levels via electrodermal activities on the skin. Although many biosensors provide adequate sensing performance, they still rely on uncomfortable, partially flexible systems with rigid electronics. These devices are mounted on either fingers or palms, which hinders a continuous signal monitoring. A fully‐integrated, stretchable, wireless skin‐conformal bioelectronic (referred to as “SKINTRONICS”) is introduced here that integrates soft, multi‐layered, nanomembrane sensors and electronics for continuous and portable stress monitoring in daily life. The all‐in‐one SKINTRONICS is ultrathin, highly soft, and lightweight, which overall offers an ergonomic and conformal lamination on the skin. Stretchable nanomembrane electrodes and a digital temperature sensor enable highly sensitive monitoring of galvanic skin response (GSR) and temperature. A set of comprehensive signal processing, computational modeling, and experimental study provides key aspects of device design, fabrication, and optimal placing location. Simultaneous comparison with two commercial stress monitors captures the enhanced performance of SKINTRONICS in long‐term wearability, minimal noise, and skin compatibility. In vivo demonstration of continuous stress monitoring in daily life reveals the unique capability of the soft device as a real‐world applicable stress monitor.

## Graphical abstract

Continuous, wireless monitoring of stress in daily life is enabled by the development of a fully integrated, stretchable, skin‐conformal bioelectronic that includes nanomembrane sensors and stretchable circuits for a highly sensitive galvanic skin response and temperature monitoring with negligible motion artifacts.

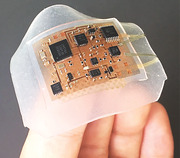

## 1 Introduction

Chronic stress is one of the significant factors causing serious health complications, such as irritability, depression, cardiovascular disease, and Alzheimer's disease.^[^ ^1^, ^2^ ^]^ Traditionally, surveys and questionnaires have been widely used to assess stress levels,^[^ ^3^, ^4^ ^]^ which is however purely subjective. In addition, a magnetic resonance imaging technique^[^ ^5^, ^6^ ^]^ has been used to measure stress. Although it is a quantitative measure, this method has big limitations of the costly process, dicrete measurement, and limited access. Recently, electrodermal activity, the variation in skin conductance and also known as galvanic skin response (GSR), has been of great interest due to the quantifiable measure of sympathetic arousal and cognitive states which are triggered along with various stressors.^[^ ^7^, ^8^, ^9^, ^10^ ^]^ GSR sensors can monitor stress activities by detecting skin conductance changes that result from the variation of the ionic permeability of sweat gland membranes.^[^ ^11^ ^]^ Typically, GSR data are measured by gel‐covered metal electrodes, mounted at near maximal concentrations of eccrine sweat glands, such as palm of the hand or fingertips. Such measurement system has limitations of device locations that can bother daily activities and motion artifacts caused by wires and gels. Recent advancements in flexible electronics have enabled wireless wearable devices that can be mounted on other locations, including foot, arm, and wrist.^[^ ^12^, ^13^, ^14^, ^15^ ^]^ However, these devices still rely on rigid metals and multiple electronic components with bulky plastic packaging and prevent the conformal skin‐to‐device interface, resulting in the use of a tightly worn band or aggressive tapes. More recent works^[^ ^16^, ^17^, ^18^, ^19^ ^]^ show some examples leveraging soft materials, stretchable designs, and skin integrations of wearable systems. However, there has been no report on the development of a fully integrated, stretchable, and wireless device for ambulatory, continuous stress monitoring in daily life (Table S1, Supporting Information).

Here, this paper reports a wireless, nanomembrane‐based SKINTRONICS that has an exceptionally small form factor for continuous monitoring of stress on either wrist or shoulder in daily life. Unlike the conventional stress monitors, our device is ultrathin, lightweight, and highly soft like a human skin, which results in a comfortable, unobtrusive mounting on the skin for continuous stress assessment. The multilayered, nanostructured device consists of a pair of skin‐conformal thin‐film sensors and stretchable membrane wireless circuit, together integrated on a soft elastomeric membrane. A comprehensive study of computational and experimental mechanics provides a key design guideline to achieve mechanical flexibility and stretchability of the device on wearable applications. The combination of GSR and temperature sensors removes any unwanted signal fluctuation caused by skin‐temperature change, while the wireless, intimate contact of the entire device on the skin provides negligible motion artifacts. Finally, a pilot study with human subjects demonstrates the device feasibility in a portable, continuous monitoring of stress in daily life, which shows the great potential of SKINTRONICS as a next‐generation, wearable stress monitor.

## 2 Results and Discussion

### 2.1 System Overview of SKINTRONICS for Stress Monitoring

**Figure 1:**
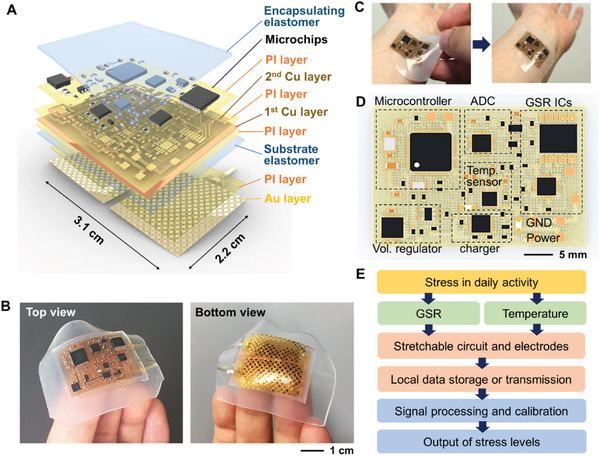
Overview of skin‐like bioelectronics (SKINTRONICS) for stress monitoring. A) Schematic illustration of the fully integrated and multilayered structure of SKINTRONICS. B) Photos showing a highly stretchable soft device on fingers with top view (left) and bottom view (right). C) Photos that capture a device placement on the wrist by simply pressing the soft membrane to the skin. D) Illustration of a stretchable circuit with multiple electronic components (annotated functional blocks). E) Flow chart capturing quantitative stress metrics from SKINTRONICS.

A comfortable, portable, and continuous monitoring of stress in daily life requires unobtrusive, ergonomic wearable device. The SKINTRONICS in this work that has an exceptionally small form factor and long‐term wearability offers such capabilities. A schematic illustration in **Figure** **1**A shows the architecture of the multilayered wearable device, including a dry, nanomembrane electrode that makes the direct contact to the skin and stretchable wireless circuit with miniaturized chips for a portable stress monitoring. The extremely low‐modulus elastomer coats and permeates the device layers to provide the natural adhesion to the skin as well as to serve as the structural support for the stretchable platform. Therefore, the integrated electrodes in the SKINTRONICS can simply be laminated onto the application site and maintain a robust and conformal adhesion to the body without requiring aggressive adhesives, bandages, or tapes.^[^ ^20^, ^21^ ^]^ Photos in Figure 1B capture a fabricated SKINTRONICS that is ultrathin (<5 mm), highly soft (effective moduli < 50 kPa), and lightweight (<7 g): top view showing an elastomer‐enclosed stretchable circuit while bottom view showing a pair of exposed skin‐mounted electrodes. The wireless wearable systems make an intimate contact to the skin (wrist) by simply pressing the device with a hand (Figure 1C). Overview of the electronic circuit design is described in an illustration (Figure 1D), including key components of GSR integrated circuits (ICs), microcontroller, analogue‐to‐digital converter (ADC), and temperature sensor (details in Figure S1, Supporting Information). The variation of skin conductance is measured by a pair of electrodes at the bottom side of circuit. A digital potentiometer, linked as a part of the Wheatstone bridge, actively controls the resistance according to a subject's skin for sensitive GSR measurement (Figure S2, Supporting Information). A digital thermistor (TMP116) measures the skin temperature with an accuracy of 0.2 °C. Figure 1E summarizes the overall flow to quantify stress levels in daily life via monitoring of GSR and temperature data. The GSR and temperature signals are recorded in a flash drive of microcontroller chip and extracted after measurement. In the detection of stress levels, the temperature sensor provides compensation of an undesired fluctuation of GSR due to the change of body temperature of a user.

### 2.2 Study of the Device Fabrication, Structural Design, and Mechanical Behavior

**Figure 2:**
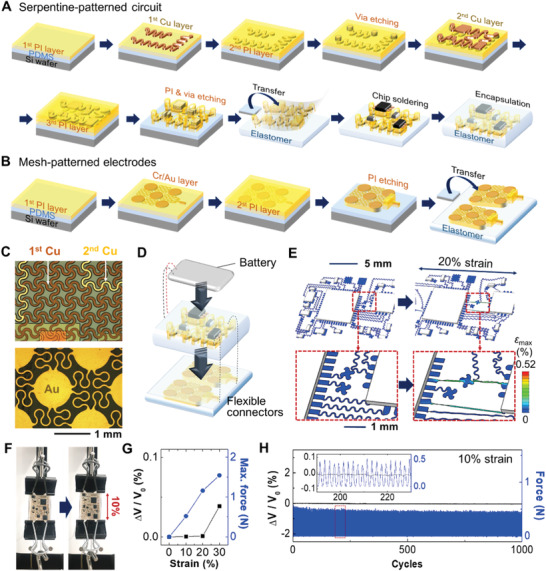
Study of the device fabrication, structural design, and characterization of mechanical behavior. A) Illustration of the fabrication steps of a stretchable nanomembrane circuit. B) Illustration showing the fabrication steps of a mesh‐patterned electrode. C) Optical microscope images of an open‐mesh, stretchable circuit with multilayers (top) and a stretchable electrode (bottom). D) Illustration showing the integration of a pair of electrodes (bottom) with a circuit (middle) and a rechargeable battery (top). Flexible microfilms make connections between the electrodes and the circuit, while the battery is attached by small magnets. E) Results of computational mechanics modeling that captures 10% tensile stretching without a mechanical fracture (scale bar: maximum principal strain). F) Experimental setup that applies a cyclic tensile strain to the device. G) Electrical measurement of voltage change upon tensile strains, showing negligible change upto 20%. H) Result of a cyclic mechanical loading on the device. 1000 cycles with 10% tensile strain has a negligible effect to the device. Inset shows the magnified view of signals changes during the cyclic loading.

The all‐in‐one, portable system, developed in this work, includes two core components: stretchable, wireless circuit (**Figure** **2**A) and mesh‐patterned nanomembrane electrode (Figure 2B). The device fabrication utilizes multiple sequential techniques, including a microfabrication process to construct necessary patterns with photolithography, metallization, transfer printing, and integration of chip components. Details of the fabrication of stretchable circuit and electrodes appear in the Experimental Section. The wireless circuit has two metal layers for ground plane and chip interconnection (top, Figure 2C). The open‐mesh, serpentine structure provides mechanical stretchability to the circuit, while isolating applied strain to the solid chips (details of the multilayered circuit design in Figure S3, Supporting Information). An optical microscope picture (bottom, Figure 2C) captures an electrode's pattern, composed of Au islands and meander interconnects, which allows over 50% stretchability and 30% areal coverage to the skin to maintain an adequate contact impedance. The electrodes are connected to the circuit via a flexible, conductive film, followed by the assembly of a lithium‐ion polymer battery with a switch (Figure 2D). The thin‐film enabled, low‐profile wearable device has about six times smaller size in volume than the most recent design of an integrated wearable GSR device.^[^ ^22^ ^]^ A computational mechanics modeling helps to design a stretchable mechanical structure for the integrated circuit. Figure 2E captures the finite element analysis (FEA) result that estimates an applied tensile strain of 20% and corresponding calculation of the minimal maximum principal strain on Cu interconnects (Cu fracture strain: 5%).^[^ ^23^, ^24^ ^]^ An experimental study of mechanical reliability in Figure 2F validates the endurance of the circuit upon cyclic tensile loading. Measured electrical voltage shows negligible changes up to 20% tensile strains (Figure 2G); after 20%, a possible deformation occurs on metal interconnects. A cyclic mechanical loading test in Figure 2H proves the device's stability up to 1000 cycles. The mechanical characteristics of the mesh‐patterned electrode were validated by our recent work,^[^ ^25^ ^]^ showing 30% uniaxial stretchability up to 400 cycles. This structural layout in the electrode and circuit ensures robust operation at strain levels beyond those that can be tolerated by the skin (10–20%).^[^ ^26^ ^]^ Overall, this study captures the device feasibility for practical use in stress monitoring on a user's skin during daily activities. It should be noted that the direct strain from skin deformation will be adsorbed by two layers of the mesh electrode and soft elastomer, which offers minimal transfer of strains to the circuit on top of the device (Figure 1A). Multi‐hour‐long functions of the SKINTRONICS are further validated in Section 2.5.

### 2.3 Characterization of the Device Performance in Comparison with Commercial Devices

**Figure 3:**
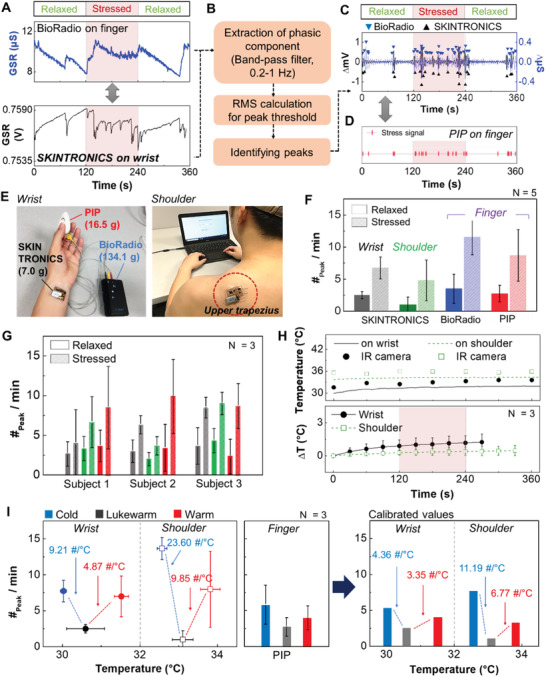
Characterization of the device performance in comparison with commercial devices. A) Comparison of raw GSR data between a commercial device (BioRadio, top) on finger and SKINTRONICS on wrist (bottom). Red squares indicate the time when stress is induced. B) Flow chart to identify phasic signals from the raw GSR. C) Comparison of identified peaks between two devices. D) Stress peaks measured by a commercial stress tester (PIP) on finger. E) Photos showing three devices to measure GSR simultaneously on wrist and finger (left) and GSR recording with SKINTRONICS on shoulder (left upper trapezius). Total weights of each device are 7.0 g (including the battery weight of 2.7 g), 16.5 g, and 134.1 g for SKINTRONICS, PIP, and BioRadio, respectively. F,G) The number of identified stress peaks per minute on different body locations and measured data from three human subjects. Error bars indicate the standard deviation. H) Comparison of temperature recording between SKINTRONICS and of IR camera (top) and temperature deviation during GSR recording on the skin (bottom). I) Measured number of stress peaks according to the change of skin temperature without external stress (left) and calibrated data set on wrist and shoulder (right).

GSR, also known as electrodermal activity, is the measure of skin conductance change, caused by human body sweating that is regulated by the autonomic nervous system. Since other factors such as temperature variation and the amount of sweat generation influence the change of GSR,^[^ ^27^, ^28^, ^29^ ^]^ the SKINTRONICS developed in this work is designed to monitor both GSR and skin temperature simultaneously. **Figure** **3**A compares the measured GSR data from a commercial device (BioRadio; top graph) and SKINTRONICS (bottom graph). The biggest difference between the two devices is the location of electrode placement; BioRadio requires the electrodes to be attached on two fingers, whereas the SKINTRONICS’ embedded stretchable electrodes are designed to seamlessly contact the wrist or the upper trapezius (shoulder), which are two of the more desirable positions for measurement of both chronic and acute stress levels.^[^ ^14^ ^]^ For performance comparison, both devices were simultaneously employed to measure the GSR of a subject under mentally relaxed and stressed states from solving arithmetic problems. For data interpretation (Figure 3B), raw GSR data were band‐passed through frequencies between 0.2 and 1 Hz to extract the phasic component and to reduce the high frequency noise.^[^ ^8^ ^]^ Next, the distribution of the peaks that are above the root‐mean‐square (RMS) threshold of the baseline phasic wave was determined.^[^ ^30^ ^]^ Figure 3C summarizes the identification of the GSR peaks from both BioRadio and SKINTRONICS, showing the high correlation in the peak distribution. For additional validation, we also compared the measured data with another device (Figure 3D), namely, PIP stress monitor, which is a handheld, consumer GSR monitoring product.^[^ ^31^ ^]^ Figure 3E compares the device form factor, mounting location, and weight of all three devices and clearly depict the advantages of SKINTRONICS with its minimal weight (7 g), compactness, and ability to be worn comfortably on the wrist or the shoulder. Details of GSR data acquired by SKINTRONICS from the shoulder location appear in Figure S4 (Supporting Information), showing the same peak patterns observed in other two commercial devices. The summary of measured stress peak data from the three devices shows that the SKINTRONICS on the wrist, despite not being attached to the fingers, is clearly capable of detecting the stressed conditions from three human subjects, where the average number of peaks during the stressed conditions is twice as high or higher as that of the relaxed conditions (Figure 3F,G). The ratio of the average number of peaks between stressed and relaxed states for SKINTRONICS is 2.69 and 4.83 for the wrist and the shoulder, respectively, comparable to BioRadio (3.31) and PIP (3.17). Such differences in the number of peaks are expected since the difference of the sweat gland densities across the fingers, the wrist, and the shoulder contributes to varying levels of GSR data.^[^ ^32^, ^33^ ^]^

Next, the effect of thermoregulation, which is related to the physiological arousal and sweat production, on GSR is investigated using SKINTRONICS. Figure 3H shows the sensitivity of the skin‐wearable device in temperature recording, which is highly comparable to a commercial infrared (IR) camera (E8, FLIR); the deviation between the two devices is ≈2 °C, which is within the camera's error range (±2 °C). Sequential thermographs captured by the IR camera for temperature validation of before and after SKINTRONICS applications appear in Figure S5 (Supporting Information). **Figure** 3I indicates that the changes in body temperature have an effect on the number of peaks even in the absence of stress and verifies the results of the prior studies that revealed the influence of temperature on the increased phasic GSR components.^[^ ^34^, ^35^ ^]^ Furthermore, simultaneous GSR measurements with three devices during relaxed states show that the increase in peak numbers at both cold and warm conditions for SKINTRONICS is significantly higher than those from PIP in Figure 3I (left). This result suggests that the observed electrodermal activity is a product of complex physiological phenomena that depend not only on the number of sweat glands but also on the body temperature. In order to remove the effect of overestimation of stress levels, two gradients of the SKINTRONICS data, found at cold and warm temperatures, were calibrated by multiplying with the gradients found in PIP. Finally, the calibrated SKINTRONICS data that consider the effects of both application site and temperature are summarized in Figure 3I (right).

### 2.4 Assessment of Signal Quality with Body Motions

**Figure 4:**
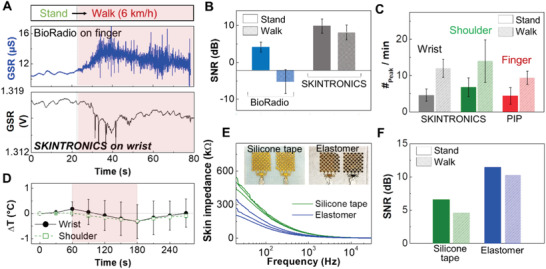
Characterization of signal quality with motions. A) Comparison of raw GSR data measured with BioRadio on finger (top) and SKINTRONICS on wrist (bottom) when a subject walks with 6 km h^−1^. B) Comparison of SNR from GSR data between two devices, capturing a significant effect of motion artifacts on BioRadio and negligible change on SKINTRONICS due to conformal lamination on the skin. Error bars show the standard deviation (*n* = 5). C) Number of measured GSR peaks that compare two skin locations with SKINTRONICS and a reference commercial device (PIP). Error bars show the standard deviation (*n* = 5). D) Temperature variation during GSR measurement with a walking subject (*n* = 3). E) The frequency‐dependent skin impedance of SKINTRONICS with two different substrates, showing the enhanced contact by the elastomer (*n* = 3). F) SNR of measured GSR data with SKINTRONICS on two types of substrates.

Existing physiological monitors are impractical for use in daily life due to various motion artifacts originating from wires, rigid electrodes, and conductive gels.^[^ ^24^, ^25^, ^36^, ^37^ ^]^ SKINTRONICS, with its compact and wearable form factor as well as the soft and dry skin–electrode interface, has the potential to enable continuous stress monitoring in daily life. Since walking is one of the most representative daily physical activities, here we compare how walking affects the GSR data qualities. A subject wearing both BioRadio on the fingers and SKINTRONICS on the wrist was asked to walk on a treadmill at 6 km h^−1^. The measured GSR data in Figure **4**A capture the comparison between with and without motions, clearly showing minimized motion artifacts from the SKINTRONICS. Noise signal elements were separated from the phasic GSR signals by the high‐pass filter with a cutoff frequency at 1 Hz and calculated for the RMS amplitude (Figure S6, Supporting Information). The summarized signal‐to‐noise ratio (SNR) of the calculated RMS in Figure 4B shows a minimized reduction with SKINTRONICS, compared to the significant reduction in the SNR with BioRadio. The robust GSR data qualities during user movement indicate that the stretchable gold nanomembrane electrodes maintain the seamless and conformal contact with the skin despite the absence of a conductive gel, validating the extremely low modulus of the elastomer substrate and the ultrathin electrode design contribute to satisfying the conformal contact criteria described in our previous work, where a multifunctional health monitor was presented.^[^ ^38^ ^]^ This result further advances the concept of conformal contact into practical wearable applications by demonstrating that the stretchable layout and extremely lightweight employed by the SKINTRONICS’ electronic module can enhance ambulatory data qualities, even on the wrist where constant motion is observed. For example, a heavier prototype device based on a nonstretchable PCB showed a large signal fluctuation during walking as well as premature mechanical failure (Figure S7, Supporting Information). Figure 4C shows the GSR peak ratios according to the motion activity, indicating a clear deviation between the two conditions. Note that the overall number of peaks on the SKINTRONICS are larger compared to the PIP. The temperature measured by SKINTRONICS gradually decreased when the subject started to walk as shown in Figure 4D, correlating with the excessive GSR counts during motion. We also investigated the contact impedance between the skin and the electrodes based on two different substrates, including a medical silicone tape (Kind Removal, 3M) and an elastomeric membrane (Figure 4E). The impedance–frequency plots clearly show that the stretchable elastomer offers enhanced contact of electrodes on the skin, compared to the more rigid silicone tape. The highly stretchable elastomer allows stable electrodes and device adhesion to the skin, evidenced by both the lower contact impedance and higher SNR than the flexible silicon tape device during walking (Figure 4F). These experimental results validate the robustness of ambulatory GSR data acquired by SKINTRONICS and verify that the low modulus elastomer substrate, stretchable designs and mechanics, and compact form factor contribute to reliable stress monitoring during walking, suggesting a significant step forward in continuous and comfortable applications.

### 2.5 Stress Monitoring in Daily Activities with SKINTRONICS

**Figure 5:**
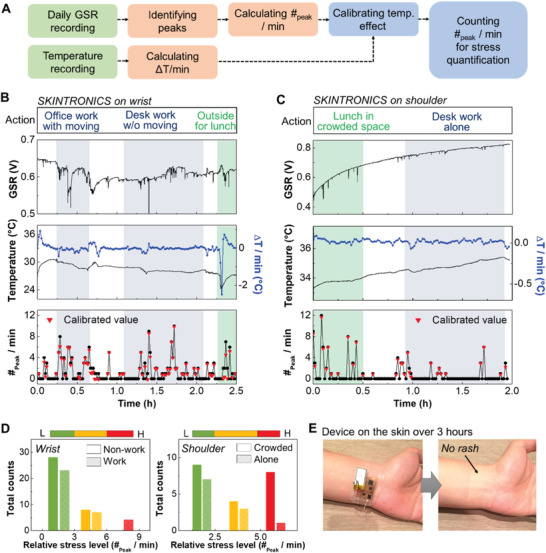
Wireless, portable, continuous stress monitoring in daily life with SKINTRONICS. A) Flow chart showing the sequential process from GSR and temperature recording to the final quantification of stress levels. B) Real‐time, continuous monitoring of GSR and temperature with SKINTRONICS on a subject's wrist during daily activities, including an office work (gray zone) with and without motions and a lunch outside (green zone). The number of peaks at the bottom shows calibrated values (red dots), considering the temperature effect. C) Recorded GSR and temperature data with SKINTRONICS on a subject's shoulder during daily activities: having a lunch in a crowded area (green zone) and doing an office work alone (gray zone). D) Summarized stress quantification and relative stress level from the wrist (left) and the shoulder (right) from the measured data in (B) and (C). E) Photos showing SKINTRONICS on the wrist for recording stress levels in daily life. The soft, gel‐free device shows no side effects to the skin after wearing over 3 h.

In this study, we demonstrated the capability of a portable, continuous stress monitoring by mounting the device on the wrist and the shoulder. The level of stress was quantified by calculating the number of GSR peaks per minute, while temperature variation was simultaneously measured to compensate for overestimation discussed previously. The flow chart in **Figure** **5**A summarizes the overall procedure of stress quantification in daily life. Figure 5B,C presents the results of measured stress levels on the wrist (Figure 5B) and the shoulder (Figure 5C). Gray zones in the graphs show the data when a subject was working on various tasks, while white zones mean resting states. The subject conducted several office tasks, such as carrying and printing documents, discussion with colleagues, and reading research articles sitting on a chair from 10 am to 3 pm. During lunch time, the subject walked into a different building and entered a crowded food court. Note that the dramatic temperature decrease was measured by the device during walking outside, which was calibrated for an accurate stress count. Also, the temperature variation on the shoulder was smaller than that on the wrist due to thick clothing. Figure 5D shows the summarized data of multihour stress levels based on the value of #peak min^−1^. The stress intensity is categorized by three levels via the relaxed/stressed ratio, measured in Figure 3F,G. Total counts of #peak min^−1^ at each level are calculated according to the specific daily activities. The highest stress levels (red bars) are shown when a subject has multiple work‐related tasks, while this subject shows the serious stress indication when exposed in a crowded space compared to being alone. Even though stress levels are subject dependent, people feel stressed during work and when surrounded by many people. Here, the wearable SKINTRONICS demonstrates the device performance as a portable, continuous stress monitor in daily life via stress level quantification. Another advantage of the soft material‐enabled device is in the skin compatibility without the use of conductive gels and aggressive tapes. Figure 5E shows that the device has no side effect (e.g., skin irritation and redness) when mounted on the skin for over 3 h. In addition, the multilayered coating of the device with polyimide and elastomer can provide the waterproof capability for a possible use during shower and/or exercise (Figure S8, Supporting Information). As a collectively comparison, **Table** **1** lists the materials and functions of recently developed wearable GSR sensors, indicating that the SKINTRONICS presented in this study is the only device with the full stretchability in both circuit and sensor and with proven stress quantification from both wrist and shoulder in daily activities.

**Table 1:** Comparison of wearable devices that measure GSR on the skin

Reference	Circuit (type)	Sensor (type)	Device location	Application	Device use time
This work	Wireless, open‐mesh patterned (stretchable)	Mesh‐patterned Au nanomembrane (stretchable)	Wrist and shoulder	Use GSR to continuously monitor stress levels in daily life	7 h
^[^ ^41^ ^]^	Wired, Cu‐PI laminated PCB (merely flexible)	Thin Cu foil (merely flexible)	Finger	Use GSR to monitor calm and active status	60 s
^[^ ^22^ ^]^	Wireless, PI‐based PCB (merely flexible)	Conventional Ag/AgCl + conductive gel (rigid)	Finger	Use GSR to monitor sleep quality	8 h
^[^ ^8^ ^]^	Wired, conventional PCB (rigid)	Conventional Ag/AgCl + conductive gel (rigid)	Finger	Use GSR to monitor emotions	40 s
^[^ ^13^ ^]^	Wireless, conventional PCB (rigid)	Metal plate in armband (rigid)	Upper arm	Use GSR to monitor sympathetic arousal	95 min
^[^ ^42^ ^]^	Wired, conventional PCB (rigid)	Ag‐Au nanowires in PI (merely flexible)	Wrist	Use GSR to evaluate the device performance	250 s
^[^ ^12^ ^]^	Wired, Raspberry Pi and Arduino board (rigid)	Cu plate + conductive gel (merely flexible)	Foot	Use GSR to evaluate the tissue condition	200 s

## 3 Conclusion

This paper reports a fully integrated, stretchable, wireless bioelectronics for a continuous, portable stress quantification in daily life. The ultrathin and lightweight device with gel‐free, nanomembrane sensors offers an ergonomic and conformal lamination on the skin for GSR and temperature recording with minimized motion artifacts. Computational modeling and experimental study of device structure and designs capture the mechanical reliability under continuous movement on skin. Comprehensive data acquisition, signal processing with simultaneous comparison of two commercial devices show the validated, enhanced performance in minimal motion artifacts and skin compatibility. The comprehensive set of studies in computational modeling, experimental mechanics, micromanufacturing, and signal processing provides the key guideline to design the wearable, stretchable stress monitor. Demonstration of the stress monitoring during a subject's daily life captures a great potential and unique capability of the SKINTRONICS as a real‐world applicable stress monitor.

## 4 Experimental Section

##### Fabrication of SKINTRONICS

SKINTRONICS has two core components, including nanomembrane stretchable electrodes and stretchable thin‐film electronic circuit. Both were developed on a Si wafer spin‐coated with polydimethylsiloxane (PDMS)/polyimide (PI) layers. For the circuit, first Cu layer was deposited and patterned by photolithography, while Cr/Au were used for electrodes. Second PI layer was coated and etched for interconnection VIA in the circuit, while the PI was etched for creating mesh patterns for the electrodes. To finish the circuit interconnects, second Cu layer was deposited and patterned as the first Cu layer, and then third PI layer additionally was coated as a protection layer of the exposed Cu layers. The laminated layers of circuit were etched as a serpentine‐shaped design and for the position of soldering. The fabricated circuit and electrodes were retrieved from the carrying PDMS/Si wafer by using water‐soluble tape (ASWT‐2, Aquasol) and placed on a soft silicone elastomer (1:2 mixture of Ecoflex 00‐30 and Gels, Smooth‐On).^[^ ^39^ ^]^ Functional microchip components were soldered on the exposed Cu layer followed by the encapsulation of elastomer.^[^ ^40^ ^]^ A rechargeable lithium‐ion polymer battery (110 mAh, LP401230, Adafruit) with a slide switch was connected to the top of circuit. Electrode part attached to the bottom side of the circuit, linking with a flexible conductive film. Details of the entire fabrication procedures appear in Note S1 (Supporting Information).

##### Computational Mechanics Modeling with FEA

FEA on the stretchable circuit was performed by using a simulation software (Abaqus, Dassault Systems). The following material properties were used in the modeling, including *E*_Cu_ = 119 GPa and *v*_Cu_ = 0.34 for Cu; *E*_PI_ = 2.3 GPa and *v*_PI_ = 0.34 for polyimide; *E*_SE_ = 7.9 kPa and *v*_SE_ = 0.49 for silicone elastomer where *E* is Young's modulus and *v* is Poisson's ratio, respectively.^[^ ^23^, ^25^ ^]^

##### Experimental Mechanics Study

Cyclic stretching experiment up to 1000 cycles was conducted to validate the estimated mechanical characteristics from the FEA, while proving the mechanical reliability of the fabricated structures. The circuit clamped on its edge was mounted on a pair of stands. A programmable motorized force gauge (M5‐5, Mark‐10) applied constant stretching cycles. Thin copper wires were connected between power pads of the device to battery for recording the change of electrical signals during cycles.

##### Measurement of GSR Data

Raw GSR signals were collected between two mesh‐patterned electrodes of SKINTRONICS, attached on the left side of inner wrist and upper trapezius site, which is one of the highly correlated site to finger GSR.^[^ ^33^ ^]^ To evaluate the quality of data measured by the SKINTRONICS, two commercial devices were utilized, including a clinical‐grade physiological monitor, BioRadio, and a portable stress monitor, PIP. To record GSR with the BioRadio, three gel‐mounted snap electrodes were attached onto the middle phalanx site of index finger and middle finger, and back of hand as a ground. For the PIP, a subject grabbed two metal plates of the device with fingers as stated by the device instruction, which should maintain the contact throughout the data recording. The recording was conducted in a room with a constant temperature 23 ± 1 °C. Overall, the human pilot study involved three healthy volunteers. The study followed the approved IRB (No. 00114274) from Emory University. All participants agreed and signed to the consent form to allow the experiment procedure.

##### Quantification of GSR Signals and Motion Artifacts

The phasic components of GSR were extracted from raw data by using the band‐pass filter with 0.2–1 Hz. RMS value of the phasic signal was calculated as a threshold level to detect peak variations. The peaks shown over the threshold level were defined as an arousal status of stress, counted the number of peaks within 1 min. SNR between the phasic GSR and noise including motion artifacts was separated by using band‐pass filter (0.2–1 Hz) and high‐pass filter (1 Hz), respectively. RMS value of each signal was calculated, and then put into the following equation

(1) $$\begin{array}{c} \mathrm{SNR}\;\left(\mathrm{dB}\right)=10\mathrm{lo}g_{10}\left(\frac{\mathrm{RMS}\_\mathrm{signal}}{\mathrm{RMS}\_\mathrm{noise}}\right) \end{array}$$

##### GSR Wireless and Flash Memory Implementations

A Bluetooth microcontroller (nRF52832, Nordic Semiconductor) was used for data acquisition, storage, and transmission. Two implementations were prepared: 1) for short‐term monitoring, Bluetooth LE protocol was used to transmit data packets in real time and 2) for long‐term monitoring, data were stored directly onto the microcontroller's flash memory. Data packets transmitted wirelessly included temperature (16‐bit) and GSR data (24‐bit), sent in packets of 50 bytes, or 10 datapoints with a connection interval of 200 ms. The sampling rate was set to 5 Hz to track changes over periods of minutes and hours. The capacity of the available flash storage was ≈262 kB, which could store 12 h of data at a sampling rate of 1 Hz or 2.4 h of data at 5 Hz. In terms of power consumption, the flash storage could record data for 7 h on a 110 mAh Li‐Po battery with an average power consumption of 66 mW. In comparison, the Bluetooth LE version depleted in 3.5 h on the same battery with an average power consumption of 130 mW. Continuous GSR and temperature data collected by the flash memory storage are presented in Figure S9 (Supporting Information).

## Conflict of Interest

Georgia Tech has a pending US patent application regarding the work presented in this paper.

## Supporting information

Supporting Information
