# Supplementary material for: Fully Integrated, Stretchable, Wireless Skin‐Conformal Bioelectronics for Continuous Stress Monitoring in Daily Life
Source: Adv Sci (Weinh). 2020 Jun 15;7(15):2000810. doi: 10.1002/advs.202000810 (PMC7404159; doi:10.1002/advs.202000810)
Supplement: Supplementary file 1 — Supporting Information [file ADVS-7-2000810-s001.pdf]

© 2020 Wiley-VCH GmbH

ADVANCED SCIENCE
Open Access

Supporting Information

for *Adv. Sci.,* DOI: 10.1002/advs.202000810

Fully integrated, stretchable, wireless skin-conformal bioelectronics for continuous stress monitoring in daily life

*Hojoong Kim, Yun-Soung Kim, Musa Mahmood, Shinjae Kwon, Nathan Zavanelli, Hee Seok Kim, You Seung Rim, Fayron Epps, and Woon-Hong Yeo**

Supporting Information

Fully integrated, stretchable, wireless skin-conformal bioelectronics for continuous stress monitoring in daily life

*Hojoong Kim, Yun-Soung Kim, Musa Mahmood, Shinjae Kwon, Nathan Zavanelli, Hee Seok Kim, You Seung Rim, Fayron Epps, and Woon-Hong Yeo**

Dr. H. Kim, Dr. Y.-S. Kim, M. Mahmood, S. Kwon, N. Zavanelli, Prof. W.-H. Yeo
George W. Woodruff School of Mechanical Engineering, Institute for Electronics and Nanotechnology, Georgia Institute of Technology, Atlanta, GA 30332, USA

Dr. H. Kim, Prof. Y. S. Rim
School of Intelligent Mechatronics Engineering, Sejong University, Seoul 05006, Republic of Korea

Prof. H. S. Kim
Department of Mechanical Engineering, University of South Alabama, Mobile, Alabama 36608, USA

Prof. F. Epps
Nell Hodgson Woodruff School of Nursing, Emory University, Atlanta, Georgia 30322, USA

Prof. W.-H. Yeo
Wallace H. Coulter Department of Biomedical Engineering, Parker H. Petit Institute for Bioengineering and Biosciences, Institute for Materials, Neural Engineering Center, Institute for Robotics and Intelligent Machines, Georgia Institute of Technology and Emory University, Atlanta, GA 30332, USA

E-mail: whyeo@gatech.edu (W.-H. Yeo)

Note S1. Fabrication process of SKINTRONICS.

1.1 Fabrication of circuit

1) Spin-coat PDMS (4:1 base-curing-agent ratio) on a Si wafer at 4000 RPM for 30 s.
2) Oxygen plasma treatment on PDMS surface for 8 s.
3) Spin-coat 1st polyimide layer (PI, PI-2610, HD MicroSystems) at 2000 RPM for 60 s.
4) Soft bake at 100 °C for 5 min and hard bake at 250°C for 1 h.
5) Deposit 0.5 µm thickness of Cu by sputtering.
6) Spin-coat photoresist (PR, Microposit SC1813, MicroChem) at 3000 RPM for 30 s, and soft bake at 100°C for 5 min. Align with a photomask and expose UV light with intensity of 15 mJ/cm^2 for 12 s and develop with a developer (MF-319, MicroChem).
7) Etch Cu with Cu etchant (APS-100, Transene) and remove remaining PR with acetone, rinse with IPA and DI water.
8) Spin-coat 2nd PI layer (PI-2545, HD MicroSystems) at 2000 RPM for 60 s, and soft bake at 100°C for 5 min. Hard bake at 240 °C for 1 h in a vacuum oven.
9) Spin-coat PR (AZ P4620, Integrated Micro Materials) at 2000 RPM for 30 sec, and soft bake at 90°C for 4 min. Photolithography exposing UV light with intensity of 15 mJ/cm^2 for 100 s. Develop with a developer (AZ-400K, Integrated Micro Materials) diluted with DI water (AZ-400K:DI water = 1:4).
10) Etch for via hole with reactive ion etcher (RIE) at 200 W, 150 mTorr, and 20 sccm of oxygen for 15 min. Rinse with acetone, IPA, and DI water.
11) Deposit 2 µm thickness of 2nd Cu by sputtering.
12) Spin-coat PR (AZ P4620) at 1500 RPM for 30 s, and soft bake at 90°C for 4 min. Photolithography exposing UV light with intensity of 15 mJ/cm^2 for 120 s and develop.
13) Etch exposed Cu with Cu etchant. Remove PR with acetone, and rinse with IPA and DI water.
14) Spin-coat 3rd PI layer (PI-2610) at 3000 RPM for 60 s. Soft bake at 100°C for 5 min and hard bake at 240°C for 1 h in a vacuum oven.
15) Spin-coat PR (AZ P4620) at 900 RPM for 30 sec, and soft bake at 90°C for 4 min. Photolithography exposing UV light with intensity of 15 mJ/cm^2 for 160 s and develop.
16) Etch exposed PI with RIE at 200 W, 150 mTorr, and 20 sccm of oxygen for 30 min. Remove remaining PR with acetone, and rinse with IPA and DI water.
17) Peel off the microfabricated circuit with water-soluble tape (ASWT-2, Aquasol) from the PDMS/Si wafer and put on a 1 mm thickness of silicone elastomer (1:2 mixture of Ecoflex 00-30 and Gels, Smooth-On). Remove tape with DI water.

18) Mount microchip components with screen-print low-temperature solder paste (alloy of Sn/Bi/Ag (42%/57.6%/0.4%), ChipQuik Inc.). Bake solder at 170 °C for 2 min.

19) Download firmware and flash a device through program line connected to circuit with magnetic cubes.

20) Etch exposed PI with reactive ion etcher (RIE) at 200 W, 150 mTorr, and 20 sccm of oxygen for 20 min.

1.2 Fabrication of electrodes

1) Spin-coat PDMS (4:1 base-curing-agent ratio) on a Si wafer at 4000 RPM for 30 s.

2) Oxygen plasma treatment on PDMS surface for 8 s.

3) Spin-coat 1st polyimide layer (PI-2610) at 2000 RPM for 60 s.

4) Soft bake at 100 °C for 5 min and hard bake at 250°C for 1 h.

5) Deposited Cr/Au by sputtering (5/200 nm).

6) Spin-coat PR (SC1813) at 3000 RPM for 30 s, and soft bake at 100°C for 3 min. Photolithography exposing UV light with intensity of 15 mJ/cm^2 for 12 s and develop with a developer (MF-319).

7) Etch Cr/Au by etchant (Chrome Mask Etchant 9030 and GE-8110, Transene) and remove remaining PR with acetone, rinse with IPA and DI water.

8) Spin-coat 2nd PI layer (PI-2545) at 2000 RPM for 60 s, and soft bake at 100°C for 5 min. Hard bake at 240 °C for 3 h in a vacuum oven.

9) Spin-coat PR (AZ P4620) at 2000 RPM for 30 sec, and soft bake at 90°C for 4 min. Photolithography with exposing UV light with intensity of 15 mJ/cm^2 for 100 s. Develop with a developer

10) Etch exposed PI except protection layer with RIE at 200 W, 150 mTorr, and 20 sccm of oxygen for 15 min. Remove remaining PR with acetone and rinse with IPA and DI water.

11) Peel off the microfabricated electrodes from the PDMS/Si wafer with water-soluble tape and put on the elastomer. Remove tape with DI water.

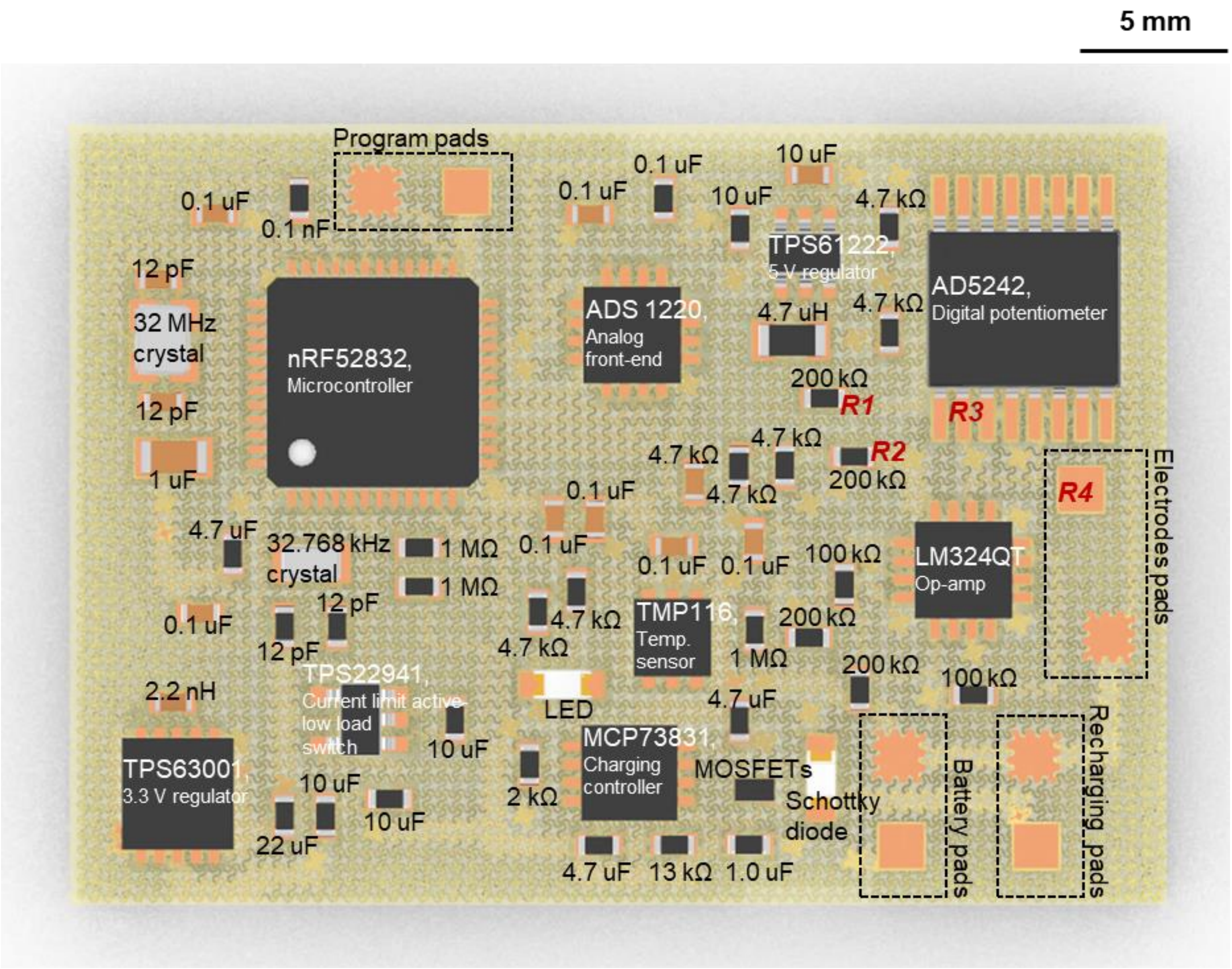

Figure S1. Top-view illustration showing chip components arrangement on SKINTRONICS. R1-R4 represent the element of the Wheatstone bridge.

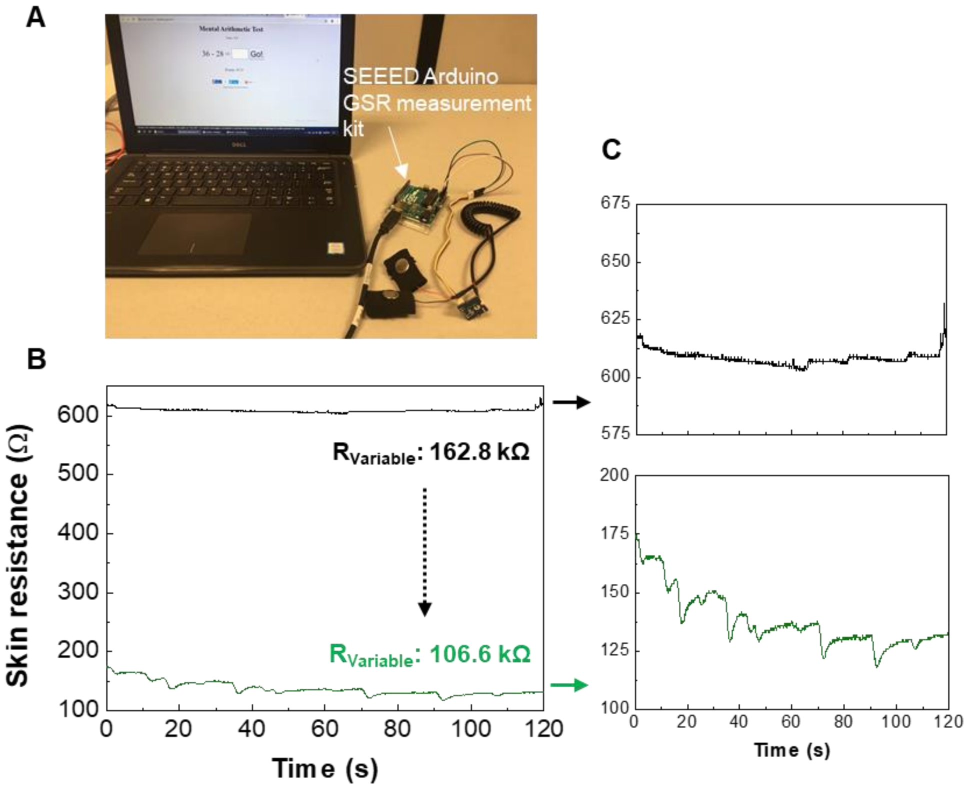

Figure S2. A) Stress experiment of GSR measurement using a commercial SEEED Arduino kit. B) The variable resistor in the circuit allows for sensitive GSR measurement regardless of the electrical resistance of any subject's skin by actively adapting its value to balance to the Wheatstone bridge circuit. C) Magnified graphs before and after adjustment of skin resistance by variable resistor.

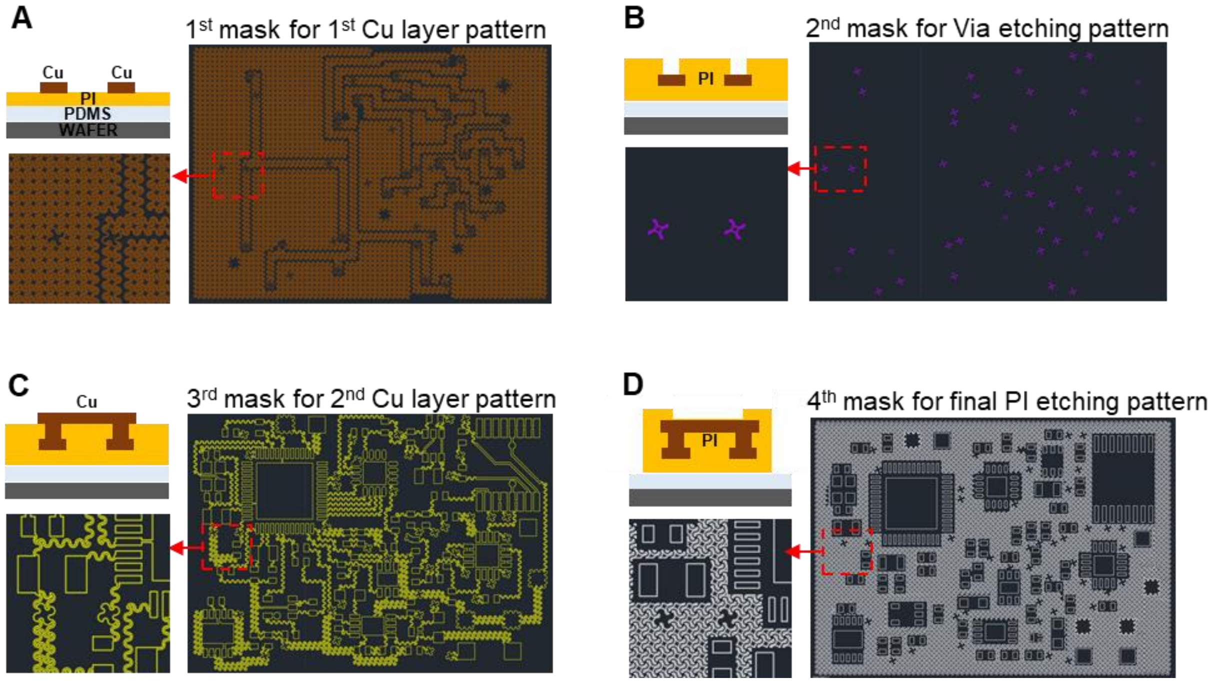

Figure S3. Mask designs for each photolithography process. Photomask patterns and resulting layer structures for A) 1st Cu layer on 1st PI layer, B) VIA etching after 2nd PI coating, C) 2nd Cu layer, and D) PI etching to define serpentine mesh layout and to expose Cu pads for soldering as well as electrodes and battery connection.

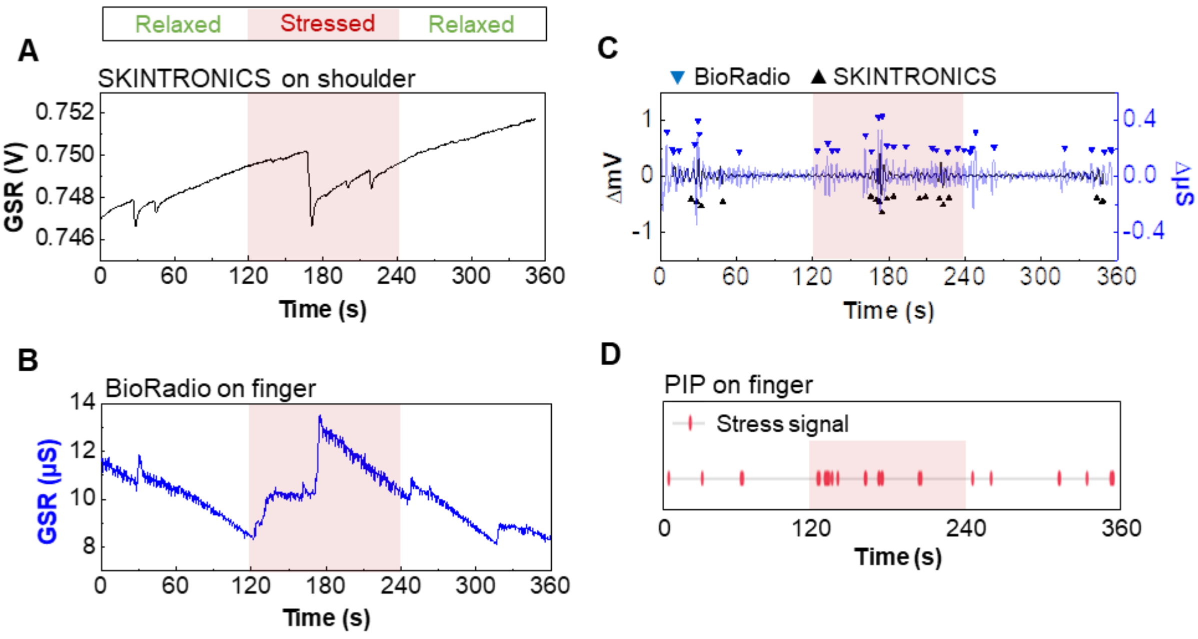

Figure S4. A) GSR signal measured on the shoulder with SKINTRONICS. C) Peak identification and comparison with B) BioRadio and D) PIP.

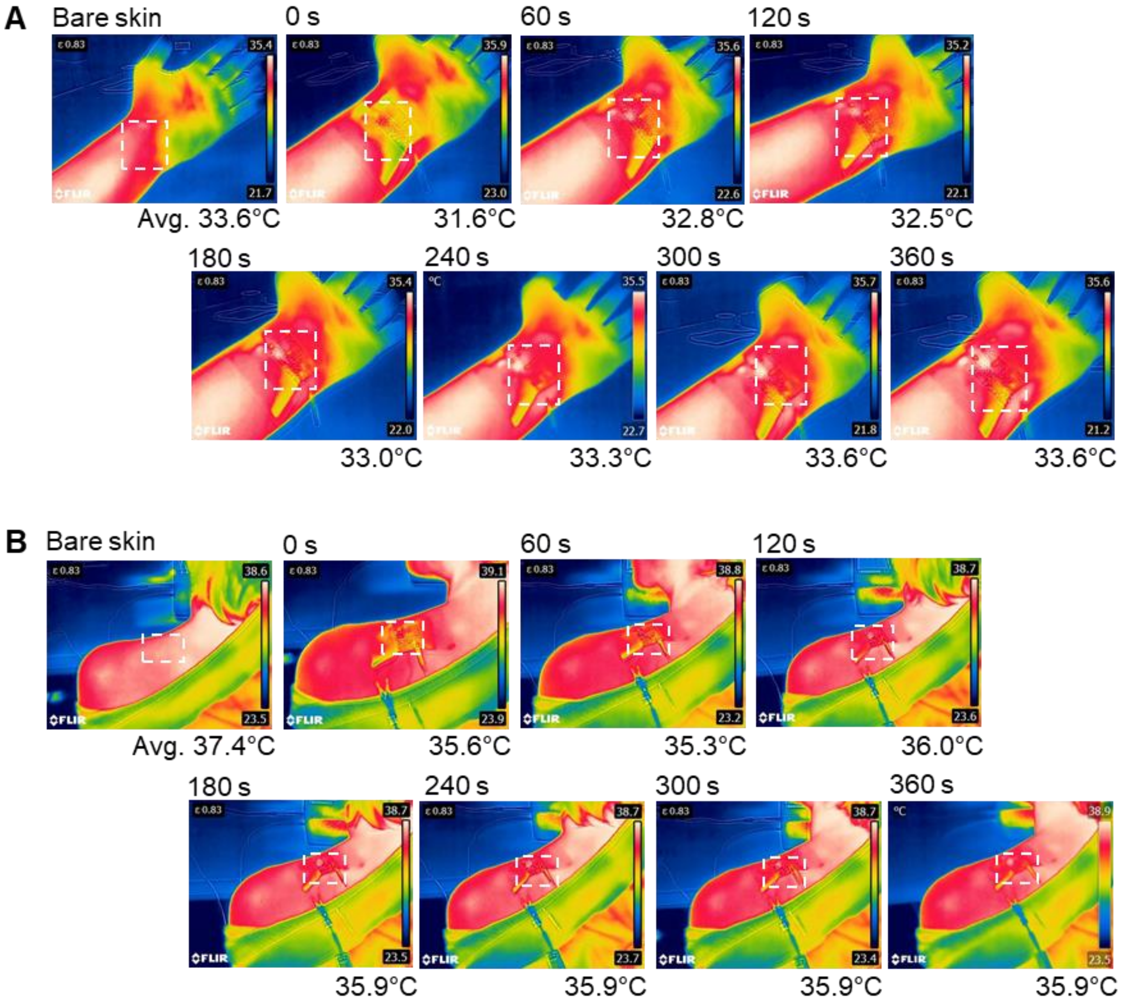

Figure S5. IR camera images capturing skin temperature with SKINTRONICS attached on the A) wrist and B) shoulder. Average temperature was calculated in the area inside white square. The average temperature gradually increased after attaching the device on the skin.

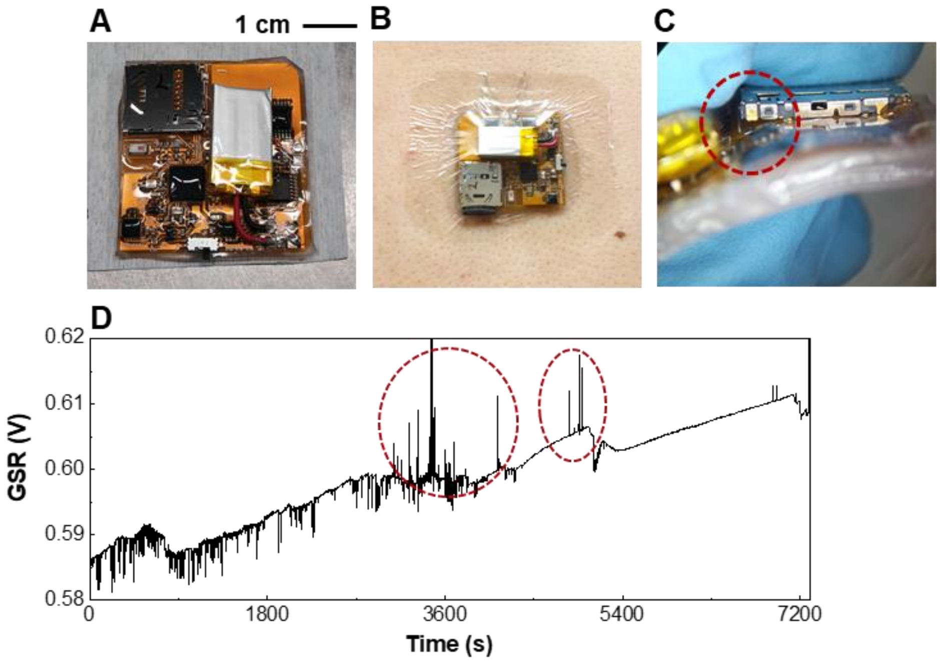

Figure S6. A) Prototype of SKINTRONICS fabricated on flexible circuit board and B) placed on the body covered with Tegaderm patch. Data was stored in the small SD card at early version of SKINTRONICS. C) However, due to its large footprint and rigid construction, the card slot easily detached from the circuit while being attached to the body. D) GSR measurement on the shoulder with prototype of SKINTRONICS for 2 h in an ambulatory setting. Large motion artifacts were observed during movement (dashed red circles) due to temporary loss of electrode-to-skin contact.

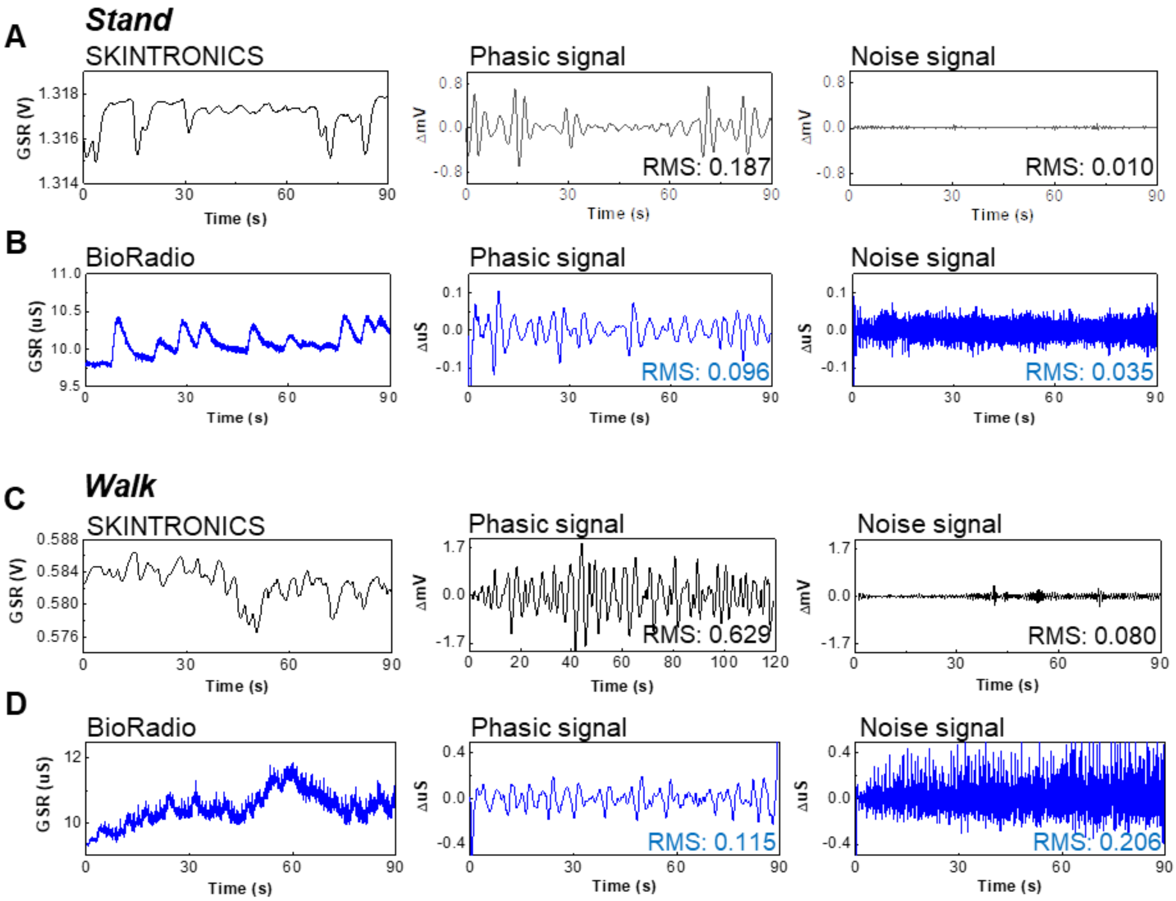

Figure S7. GSR data and their signal processing signals measured from SKINTRONICS and BioRadio with A-B) stand and C-D) walk status, respectively. A significant increase in the RMS value of noise signal from BioRadio was observed as the subject began to walk.

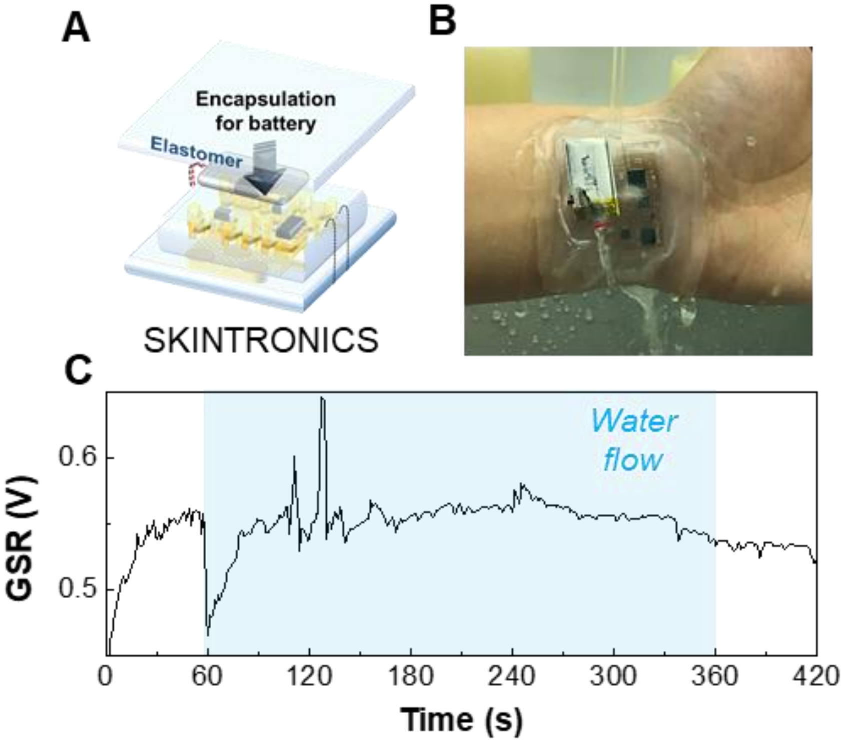

Figure S8. Waterproof capability of SKINTRONICS. A) An additional elastomeric layer encapsulated the battery and the circuit components. B) Photo of fully insulated SKINTRONICS on the wrist under flowing water. C) A stream of water was applied to the device for 5 min during GSR measurement. The device maintained its functionality even in the presence of flowing water without noticeable compromise in its attachment to the body and the measured signals, suggesting the feasibility for GSR measurements in excessively moist settings.

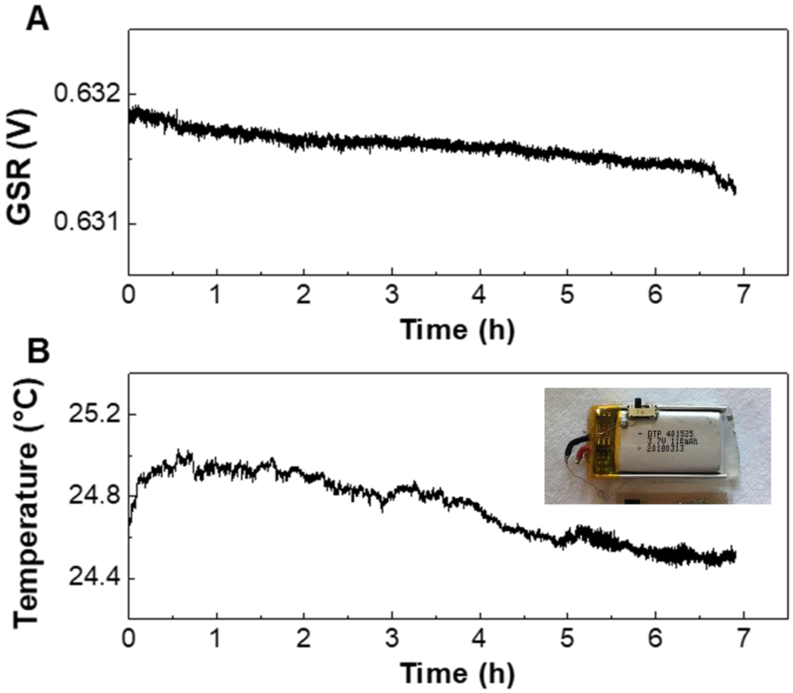

Figure S9. Maximum operation time of SKINTRONICS. A) GSR and B) temperature signals can be recorded for 7 hours using the on-board flash memory. Inset picture shows the 110 mAh lithium-ion polymer battery with an integrated slide switch.

Table S1. Comparison of stretchable sensors and circuit systems.

Ref	Group (year)	Circuit type	Electrodes type	Device type	Sensing target	Application
This work	Yeo group (2020)	Open-mesh patterned PI/Cu/PI/Cu/PI on elastomer	Mesh-patterned PI/Cr/Au/PI on elastomer	Integrated	GSR, temperature	Daily stress monitoring
[1]	Rogers group (2019)	N/A	Serpentine-patterned Cr/Au on elastomer	Separated	EMG, ECG, EEG	Prosthetic control and cognitive monitoring
[2]	Rogers group (2018)	Non-patterned PI/Cu/PI/Cu	Serpentine-patterned PI/Cu/PI/Cu/PI on elastomer	Integrated	Temperatur e, Blood flow	Shunt failure monitoring
[3]	Rogers group (2018)	Non-patterned PI/Cu on adhesive	Serpentine-patterned PI/Cr/Au/PI/ on elastomer	Integrated	Temperature	Thermal characterization of human skin
[4]	Rogers group (2017)	N/A	Serpentine-patterned PI/Cu/PI on elastomer	Separated	Temperature	Thermal characterization of human skin
[5]	Yu group (2019)	N/A	Open-mesh patterned PI/Au/IZO/PI on elastomer	Separated	Strain	Robotic hand control
[6]	Xu group (2018)	Serpentine-patterned 4 layered PI/Cu on elastomer	Non-patterned CNT and silbione	Integrated	EMG, ECG, temperature	Robotic hand control
[7]	Xu group (2018)	N/A	Serpentine-patterned PI/Cu/Sn/transducer/ Cu/PI on elastomer	Separated	Blood pressure	Central blood pressure waveform monitoring

References

[1] L. Tian, B. Zimmerman, A. Akhtar, K. J. Yu, M. Moore, J. Wu, R. J. Larsen, J. W. Lee, J. Li, Y. Liu, B. Metzger, S. Qu, X. Guo, K. E. Mathewson, J. A. Fan, J. Cornman, M. Fatina, Z. Xie, Y. Ma, J. Zhang, Y. Zhang, F. Dolcos, M. Fabiani, G. Gratton, T. Bretl, L. J. Hargrove, P. V. Braun, Y. Huang, J. A. Rogers, *Nat. Biomed. Eng.* **2019**, 3, 194.

[2] S. R. Krishnan, T. R. Ray, A. B. Ayer, Y. Ma, P. Gutruf, K. Lee, J. Y. Lee, C. Wei, X. Feng, B. Ng, *Sci. Transl. Med.* **2018**, 10.

[3] S. R. Krishnan, C. J. Su, Z. Xie, M. Patel, S. R. Madhvapathy, Y. Xu, J. Freudman, B. Ng, S. Y. Heo, H. Wang, T. R. Ray, J. Leshock, I. Stankiewicz, X. Feng, Y. Huang, P. Gutruf, J. A. Rogers, *Small* **2018**, 14, e1803192.

[4] L L. Tian, Y. Li, R. C. Webb, S. Krishnan, Z. Bian, J. Song, X. Ning, K. Crawford, J. Kurniawan, A. Bonifas, J. Ma, Y. Liu, X. Xie, J. Chen, Y. Liu, Z. Shi, T. Wu, R. Ning, D. Li, S. Sinha, D. G. Cahill, Y. Huang, J. A. Rogers, *Adv. Funct. Mater*. **2017**, 27.

[5] K. Sim, Z. Rao, Z. Zou, F. Ershad, J. Lei, A. Thukral, J. Chen, Q.-A. Huang, J. Xiao, C. Yu, *Sci. Adv.* **2019**, 5, eaav9653.

[6] Z. Huang, Y. Hao, Y. Li, H. Hu, C. Wang, A. Nomoto, T. Pan, Y. Gu, Y. Chen, T. Zhang, W. Li, Y. Lei, N. Kim, C. Wang, L. Zhang, J. W. Ward, A. Maralani, X. Li, M. F. Durstock, A. Pisano, Y. Lin, S. Xu, *Nat. Electron.* **2018**, 1, 473.

[7] C. Wang, X. Li, H. Hu, L. Zhang, Z. Huang, M. Lin, Z. Zhang, Z. Yin, B. Huang, H. Gong, S. Bhaskaran, Y. Gu, M. Makihata, Y. Guo, Y. Lei, Y. Chen, C. Wang, Y. Li, T. Zhang, Z. Chen, A. P. Pisano, L. Zhang, Q. Zhou, S. Xu, *Nat. Biomed. Eng.* **2018**, 2, 687.
